# Deficiency of neuropeptide Y attenuates neointima formation after vascular injury in mice

**DOI:** 10.1186/s12872-023-03267-y

**Published:** 2023-05-06

**Authors:** Song Peng, Wei-qiang Wu, Lin-yu Li, Yan-chuan Shi, Shu Lin, Zhi-yuan Song

**Affiliations:** 1 Department of Cardiology, Southwest Hospital, Third Military Medical University (Army Medical University), Chongqing, China; 2 Group of Neuroendocrinology, Garvan Institute of Medical Research, 384 Victoria St, Sydney, Australia

**Keywords:** Neuropeptide Y, Restenosis, Neointimal formation, Inflammation

## Abstract

**Background:**

Restenosis after percutaneous coronary intervention (PCI) limits therapeutic revascularization. Neuropeptide Y (NPY), co-stored and co-released with the sympathetic nervous system, is involved in this process, but its exact role and underlying mechanisms remain to be fully understood. This study aimed to investigate the role of NPY in neointima formation after vascular injury.

**Methods:**

Using the left carotid arteries of wild-type (WT, NPY-intact) and NPY-deficient (NPY^−/−^) mice, ferric chloride-mediated carotid artery injury induced neointima formation. Three weeks after injury, the left injured carotid artery and contralateral uninjured carotid artery were collected for histological analysis and immunohistochemical staining. RT-qPCR was used to detect the mRNA expression of several key inflammatory markers and cell adhesion molecules in vascular samples. Raw264.7 cells were treated with NPY, lipopolysaccharide (LPS), and lipopolysaccharide-free, respectively, and RT-qPCR was used to detect the expression of these inflammatory mediators.

**Results:**

Compared with WT mice, NPY^−/−^ mice had significantly reduced neointimal formation three weeks after injury. Mechanistically, immunohistochemical analysis showed there were fewer macrophages and more vascular smooth muscle cells in the neointima of NPY^−/−^ mice. Moreover, the mRNA expression of key inflammatory markers such as interleukin-6 (IL-6), transforming growth factor-β1 (TGF-β1), and intercellular adhesion molecule-1 (ICAM-1) was significantly lower in the injured carotid arteries of NPY^−/−^ mice, compared to that in the injured carotid arteries of WT mice. In RAW264.7 macrophages, NPY significantly promoted TGF-β1 mRNA expression under unactivated but not LPS-stimulated condition.

**Conclusions:**

Deletion of NPY attenuated neointima formation after artery injury, at least partly, through reducing the local inflammatory response, suggesting that NPY pathway may provide new insights into the mechanism of restenosis.

## Introduction

Despite significant progress in prevention and treatment, ischemic heart disease due to atherosclerosis remains one of the leading causes of death [1]. Revascularization with percutaneous coronary angioplasty (PCA) and stenting adequately improves the blood supply to the heart, but leads to an increased risk of restenosis, a common and serious complication due to the damage of innermost lining of blood vessels [2]. Neointima formation refers to the new scar tissue formed by vascular surgical procedures such as angioplasty and stent placement, and is the pathological basis of restenosis; however the underlying mechanisms are not fully understood [3].

There are abundant nerve fibers in the artery wall, and arterial function is regulated by neural activity [4, 5]. Emerging evidence demonstrates that neurotransmitters, such as noradrenaline and acetylcholine, are involved in the pathogenesis of arterial diseases [6, 7]. Neuropeptide Y (NPY) is widely expressed in the central and peripheral nervous systems and plays a crucial role in food intake and energy balance [8]. In the periphery, NPY and norepinephrine are co-stored and co-released at sympathetic nerve terminals [9] which have been shown to be involved in the process of neointima formation in injured blood vessels [10, 11]. In an early study, rat carotid artery underwent balloon angioplasty, and NPY pellets that were placed next to the injured artery led to augmented angioplasty-induced neointima [12]. Shah et al. conducted wire induced endothelial denudation in the left common carotid artery of ApoE^−/−^ mice and applied a pluronic gel, containing BIBP 3226 (an NPY Y1 receptor antagonist), to the outer surface of the injured vessel for six weeks. They found that neointima formation was significantly reduced in BIBP3226-treated mice, suggesting that NPY mediates neointimal progression after artery injury in ApoE^−/−^ mice through the Y1 receptor [13]. However, conflicting findings have been shown in the literature. For example, Pesonen and colleague found that in patients with leucine 7 to proline 7 polymorphism of the preproneuropeptide Y gene, affecting plasma NPY levels, the rates of post-PCI restenosis was equal to those without this polymorphism [14]. Therefore, the physiopathological role of NPY in arterial disease, especially after vascular injury, remains controversial. Obviously, further studies are needed to understand the exact mechanism. There is evidence that NPY regulates the functions of inflammatory cells in vitro, such as monocyte chemotaxis, macrophage phagocytosis, lymphocyte proliferation were regulated by NPY [15–17]. We sought to study how NPY affected on post vascular injury-induced neointima formation via the modulation of inflammation responses.

There was a significant overlap of mouse genes with human genes, remarkably, 50% of coronary artery disease-associated pathways derived from mouse GWAS overlapped with those identified from human studies [18]. With its ease of genetic manipulation and human disease imitation, the mouse is most frequently used model for cardiovascular disease studies. In the present study, we utilized novel NPY deficient mice where NPY gene was reactivatable knockout to understand the role of NPY in neointima formation after vascular injury. Our findings suggest that NPY promotes neointima formation after arterial injury by modulating key inflammatory markers. The present study provides new insights into the pathogenesis of neointimal formation and potential new therapies for the intervention of restenosis.

## Materials and methods

### Animals

All animal care and experimental procedures were approved by the Third Military Medical University Animal Welfare and Ethics Committee and followed the National Institutes of Health Guide for the Care and Use of Laboratory Animals. Male C57BL/6 mice were purchased from the animal center of the Third Military Medical University (Chongqing, China) and used as control throughout this study. Mice carrying a reactivatable NPY knockout allele were generated at Cyagen Biosciences (Suzhou, China) by inserting a transcription-translation stop cassette flanked by loxP sites (LSL) in the first intron of the endogenous WT NPY locus. Through this genetic strategy, the expression of *NPY* was efficiently prevented by the STOP cassette and could be restored via excising the STOP cassette by Cre recombinase as described previously [19]. The NPY^LSL/LSL^ mice are viable and fertile. Early observation revealed the body weight and activity were normal in NPY^LSL/LSL^ mice, and no gross physical or behavioral abnormalities were detected. The homozygous NPY^LSL/LSL^ mice were systemically *NPY*-silenced and functionally equivalent to NPY knockout mice (referred to here after as NPY^−/−^ mice). Before the age of 7 weeks, all mice were fed a standard rodent chow. Two weeks before surgery (when the mice were 7 weeks old), the chow was changed to a Western-type diet (16% fat and 1.25% cholesterol), which was maintained throughout the experiment.

The sample size of mice for injured carotid artery morphological analysis was estimated through power analysis. Before animal experiment initiate, we found that in published research the stenosis of mice carotid artery three weeks after FeCl_3_ induced injury was 0.375 ± 0.025 [20]. Presuppose carotid artery stenosis in NPY^−/−^ mice may decrease 50%, perform power analysis with 0.05 significance and 90% power, the results indicated the sample size should be 15 per group. Meanwhile, mice number for carotid gene expression analysis was determined according to the published study as 10 per group [21]. Thus, the sample size of WT and NPY^−/−^ mice were decided as 25 per group.

### Ferric chloride-induced carotid artery injury

For experiments of ferric chloride-induced carotid artery injury, we used 7-week-old, 20 ~ 22-g-weight, male NPY^−/−^ mice and age-weight-matched male WT C57BL/6 mice. After two weeks of western-type diet feeding, these mice were performed carotid artery injury. Ferric chloride (FeCl_3_)-induced carotid artery injury in WT C57BL/6 and NPY^−/−^ mice, as previously described [22]. Mice were anesthetized by intraperitoneal injection of phenobarbital sodium (60 mg/kg). Prior to performing procedures confirm mice are at an adequate depth of anesthesia by toe pinching. The left carotid sheath was exposed through a cervical, middle longitudinal incision. Search carotid sheath through the spaces between the sternocleidomastoid muscle and omohyoid muscle or sternohyoid muscle to avoid muscle injury and reduce operating time, thereby decreasing the suffering of mice. After opening the carotid sheath and dissociating the midportion of the left carotid artery, a piece of 0.5 mm×1 mm Whatman filter paper, which was saturated with 10% FeCl_3_ solution, was placed on the artery surface for 3 min to induce vascular injury. The cervical incision was then sutured, and the mice returned to their cages. To control experiment confounders, the sets of WT C57BL/6 and NPY^−/−^ mice were performed surgeries on the same day and by the same researcher.

### Histology and immunohistochemistry

Twenty-one days after surgical manipulation, the mice were anesthetized again, and the injured carotid artery on the left side and the uninjured carotid artery on the right side were dissected carefully. Then euthanize the mice by cervical dislocation. The atrium dextrum was incised, and cold saline was perfused into the left ventricle for 5 min to eliminate blood. The carotid arteries were cut and put into optimal cutting temperature compound (OCT) then frozen in the − 80℃ refrigerator. Frozen carotid arteries were cut into 10-µm-thick sections using a cryostat (model CM3050S; Leica Microsystems, Buffalo Grove, USA). Three sections of every injured artery, equally spaced throughout the injured segment (at 250 μm intervals), were stained with hematoxylin and eosin (H&E) to evaluate neointima formation, and the results were averaged for each mouse [23].

Immunohistochemical analysis was performed using a commercial IHC kit according to the manufacturer’s instructions (ZSGB-BIO, Beijing, China). Sections were fixed in cold formalin for 10 min, rinsed with PBS three times, incubated with endogenous peroxides blockers for 10 min, rinsed with PBS, and blocked with serum corresponding to the relevant secondary antibody species for 20 min. The sections were then incubated with primary antibodies against NPY (ab30914, Abcam, Cambridge, UK), MOMA-2 (ab33451, Abcam; for macrophages) or α-smooth muscle actin (ab5694, Abcam; for smooth muscle cells) overnight at 4 °C. Isotype IgG control corresponding to the primary antibody species were used to checked for non-specific binding of the antibodies. After PBS rinsing, the sections were incubated with horseradish peroxidase-conjugated secondary antibodies for 20 min, then visualized by staining with 3,3′-diaminobenzidine substrate and counterstaining with hematoxylin. The slices were photographed using a Nikon DS-Ri2 digital camera, equipped on a Nikon ECLIPSE Ni microscope and analyzed using ImageJ software. The operators were blinded to specimen genotype when performing analyses.

### RNA isolation from carotid arteries

Three weeks after the artery injury, the mice were sacrificed and perfused with cold saline for 5 min. The injured carotid artery was then rapidly excised and stored with RNA stabilization solution (AM7021, ThermoFisher, Waltham, USA) at -20℃. Three to four carotid arteries were pooled per sample for total RNA isolation using TRIzol reagent (Tiangen Biotech, Beijing, China). RNA was reverse transcribed into complementary DNA using a cDNA synthesis kit (KR118, Tiangen Biotech) according to the manufacturer’s instructions.

### Cell culture

The mouse macrophage cell line RAW264.7 cells were purchased from ATCC (Manassas, USA) and grown in DMEM media (Gibco, Rockville, USA) supplemented with 10% fetal bovine serum (Gibco, Rockville, USA), 100 U/ml penicillin and 100 mg/ml streptomycin at 37℃ in 5% CO_2_.

### Proliferation assay

The effect of NPY on RAW264.7 cell proliferation was determined using a Cell Counting Kit-8 (CCK-8) (Dojindo, Kumamoto, Japan) assay and was performed according to the manufacturer’s instructions. The cells were grown in 96-well plates with a concentration of 1.0 × 10^4^ cells/well and cultured overnight. Then the cells were treated with 10^− 8^ M-10^− 6^ M NPY (Tocris, Bristol, UK) or its vehicle PBS for 24 h. After that, 10ul CCK8 reagent was added into every well, and the mixture incubated for another 3 h at 37℃. Cell proliferation rate was measured at 450 nm using an ELISA reader (Varioskan Flash, Thermo Scientific, Waltham, USA). The experiment was repeated at least three times for confirmation.

### Gene expression studies

The inflammatory profile was detected after NPY incubation in macrophages in the presence or absence of LPS. Briefly, mouse macrophage RAW264.7 cells were seeded in 6-well plates at a density of 6 × 10^5^ cells/ml. When cells were cultured overnight, they were pretreated with NPY at concentrations of 10^− 7^ M or PBS for 30 min and then incubated with or without LPS (10 ng/ml) (Beyotime, Shanghai, China) for 12 h. Thereafter, total RNA was isolated from the cultured macrophages using TRIzol reagent (Tiangen Biotech, Beijing, China). RNA was reverse transcribed into complementary DNA using a cDNA synthesis kit (KR118, Tiangen Biotech) according to the manufacturer’s instructions.

### Real-time quantitative polymerase chain reaction analysis

Real-time quantitative PCR (RT-qPCR) was conducted using the SYBR Green SuperReal PreMix Plus kit (Tiangen Biotech) in a C1000™ Thermal Cycler (Bio-Rad, Hercules, USA). The primer sequences of these studied genes were shown in Table 1. The expression level of target gene mRNA was calculated using the 2^−ΔΔCt^ method, which normalized the abundance of target genes with that of the internal control β-actin.

**Table 1:** Primer sequences for real-time qPCR

Gene	Forward sequence (5′-3′)	Reverse sequence (5′-3′)
IL-1β	GCAGCAGCACATCAACAAGAGC	AGGTCCACGGGAAAGACACAGG
IL-6	CTCCCAACAGACCTGTCTATAC	CCATTGCACAACTCTTTTCTCA
TGF-β1	CCAGATCCTGTCCAAACTAAGG	CTCTTTAGCATAGTAGTCCGCT
ICAM-1	CTGAAAGATGAGCTCGAGAGTG	AAACGAATACACGGTGATGGTA
VCAM-1	GACATTTACCCAGTTTACAGGC	TGACGGGAGTAAAGGTTACTTC
β-actin	GTGCTATGTTGCTCTAGACTTCG	ATGCCACAGGATTCCATACC

### Statistical analysis

Data are expressed as mean ± standard error of the mean (SEM). A 2-tailed Student’s t-test was used to compare individual groups. SPSS 11.0 software was used to carry out all statistical analysis. Differences with *p* < 0.05 were considered statistically significant.

## Results

### Successful generation of carotid artery injury models and the effect of NPY deficiency on neointima formation

**Fig. 1:**
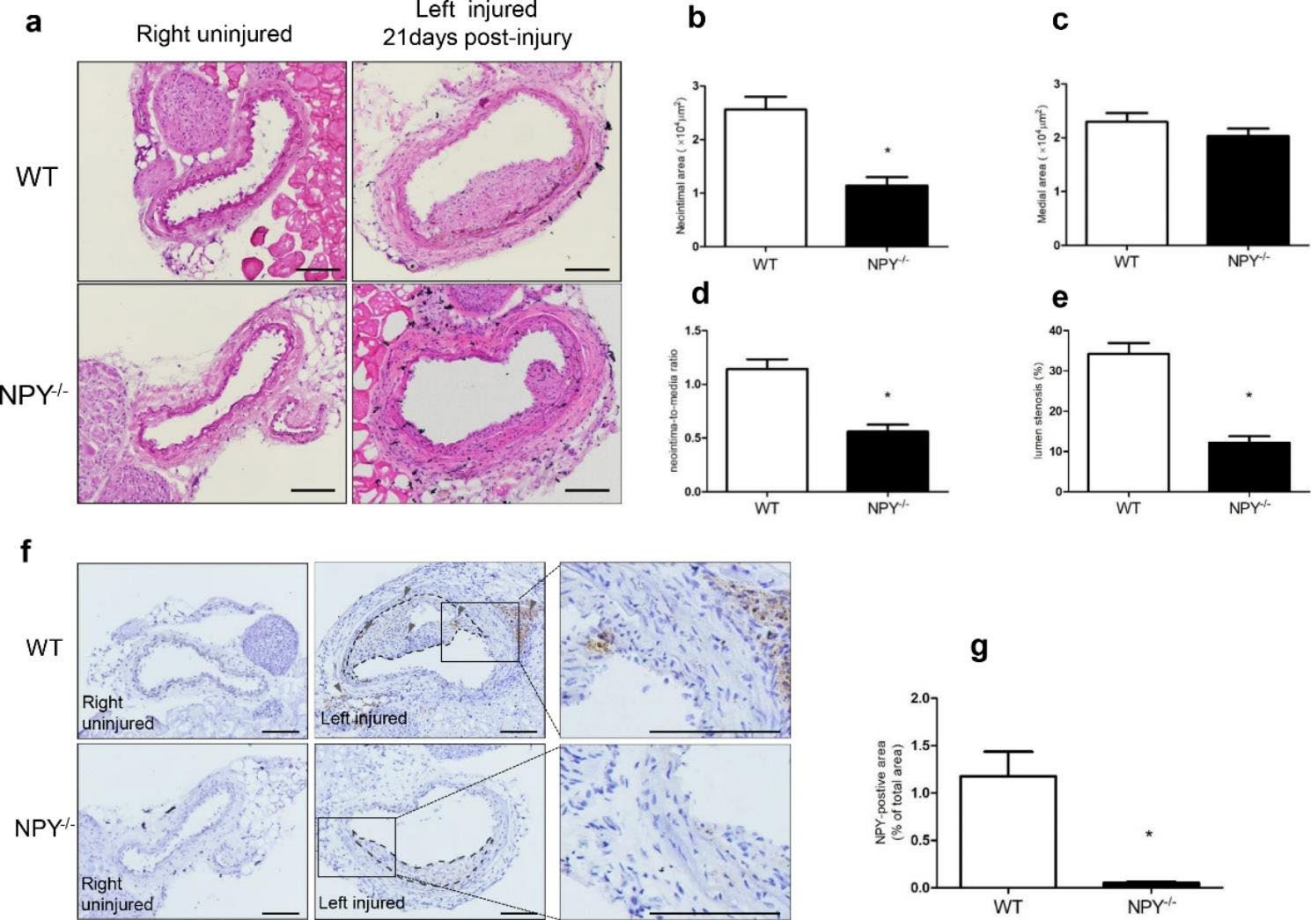
Generation of carotid artery injury model and the effect of lack of NPY on neointima formation. **a** Representative H&E staining of carotid artery sections from wild-type and NPY^−/−^ mice, 21 days after artery injury. The bar represents 100 μm. **b–e** The bar graphs show the results of morphometric analysis. The neointimal area (b), neointima/media ratio (d) and lumen stenosis (e) were significantly reduced in NPY^−/−^ mice compared to those in the WT control (n = 15 mice/group). **f** Expression of NPY in carotid arteries 21 days after injury. Representative NPY immunohistochemical staining micrographs are shown. The dotted line indicates the area of neointima. The arrowheads indicate the NPY-positive areas in the neointima, media, and perivascular tissue in the injured carotid artery of the WT mice. The bar represents 100 μm. **g** Quantification of NPY immunostaining in carotid arteries of WT and NPY^−/−^ mice 21 days after FeCl_3_ induced injury. Differences between groups were assessed by Student’s t-test, *p < 0.05

To induce neointimal formation following vascular injury, we performed FeCl_3_-induced carotid artery injury in WT and NPY^−/−^ mice. As shown in Fig. 1, in WT mice, the intima of the right uninjured carotid artery was intact, smooth and without hyperplasia; in contrast, there was marked neointimal formation on the left injured carotid artery 21days after vascular injury, resulting in luminal stenosis (Fig. 1a). In NPY^−/−^ mice, the inner wall of the uninjured carotid artery on the right was clean and unobstructed, and the injured carotid artery on the left formed a slight intimal lesion that invaded the lumen (Fig. 1a). These findings suggested that the generation of artery injury models was successful in these two types of mice. Interestingly, we observed that the size of neointima from carotid injury appeared to be smaller in NPY^−/−^ mice than in WT mice. Next, we sought to quantify the size of the neointima in the presence (WT) or absence (NPY^−/−^) of NPY to analyse the effect of NPY on neointima formation. Compared with WT mice, NPY^−/−^ mice had significantly reduced the neointimal area [(2.562 ± 0.241)×10^4^µm^2^ in WT mice versus (1.145 ± 0.156)×10^4^µm^2^ in NPY^−/−^ mice, *p* < 0.05]; the neointima/media ratio (1.142 ± 0.091 in WT mice versus 0.559 ± 0.067 in NPY^−/−^ mice, *p* < 0.05) and lumen stenosis (34.227 ± 2.691% in WT mice versus 12.212 ± 1.567% in NPY^−/−^ mice, *p* < 0.05) were also significantly reduced (Fig. 1b,d,e). These data suggest that NPY deficiency could effectively inhibit neointima formation following vascular injury. We next examined the NPY levels in the injured arteries of WT mice and compared with the contralateral side of the same WT mouse and that in NPY^−/−^ mice. The expression of NPY was not detected in the uninjured carotid artery on the right side of WT mice (Fig. 1f), whereas NPY-positive regions were present in the neointima, media and perivascular tissue of the injured left carotid artery from the same WT mice (Fig. 1f). This data indicated that NPY was upregulated in the neointima and the media as well as the perivascular tissue after the carotid artery injury, thereby participating in the neointima formation. In NPY^−/−^ mice, as expected, the NPY immunohistochemical staining was negative in both contralateral uninjured and left injured carotid arteries (Fig. 1f,g), further confirm the absence of NPY in this NPY deficient mouse model.

### The effect of NPY on the content of macrophages and vascular smooth muscle cells in neointima

**Fig. 2:**
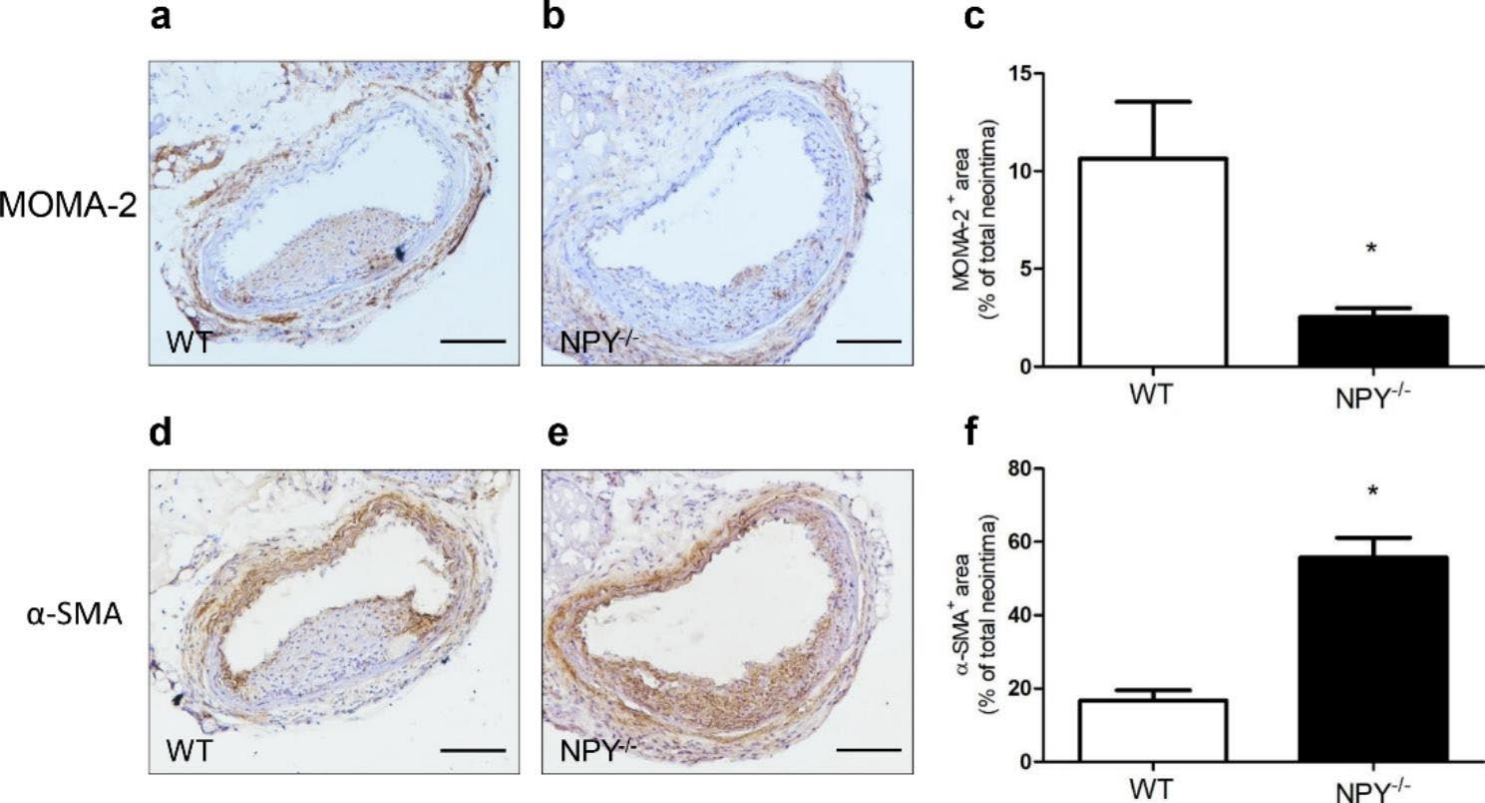
Detection of macrophages and VSMCs in the neointima. FeCl_3_-mediated carotid artery injury was performed in WT and NPY^−/−^ mice. Twenty-one days after the injury, the carotid arteries were excised for immunohistochemical staining. Representative pictures of immunostaining with anti-MOMA-2 antibody (for macrophage, a,b) and anti-a-SMA antibody (for VSMCs, d,e) are depicted. The bar represents 100 μm. MOMA-2-positive neointimal area was significantly reduced (c), and a-SMA-positive neointimal area was increased (f) in the NPY^−/−^ mice compared to that in the WT mice. Values are mean ± SEM (n = 5), *p < 0.05

Inflammation has been shown to play an important role in the process of neointima formation [24], and vascular smooth muscle cells (VSMCs) are the major cell types in the neointima [25]. To determine whether inflammatory pathways have been involved in NPY-mediated neointima formation, we assessed macrophages (MOMA-2) in the injured carotid artery sections from WT and NPY^−/−^ mice using immunohistochemistry. Meanwhile, VSMCs (α-SMA) were also detected with immunohistochemistry (Fig. 2). We found that compared to that in the WT mice, the MOMA-2-positive area of the neointima of the injured carotid artery in the NPY^−/−^ mice was significantly decreased (10.65 ± 2.91% vs. 2.53 ± 0.46% in WT and NPY^−/−^ mice, respectively; *p* < 0.05, Fig. 2c). The α-SMA-positive area of the neointima was increased in the NPY^−/−^ mice (16.65 ± 2.87% vs. 55.71 ± 5.49% in WT and NPY^−/−^ mice, respectively; *p* < 0.05, Fig. 2f). These results suggest that in the absence of NPY, the macrophage gathering was likely to be inhibited in the neointima, thereby avoiding inflammatory damage to the artery. Moreover, the enhancement of VSMCs density by the lack of NPY in the neointima may improve the stability of the vascular lesion.

### NPY influences mRNA expression of inflammatory markers and cell adhesion molecules in injured arteries

**Fig. 3:**
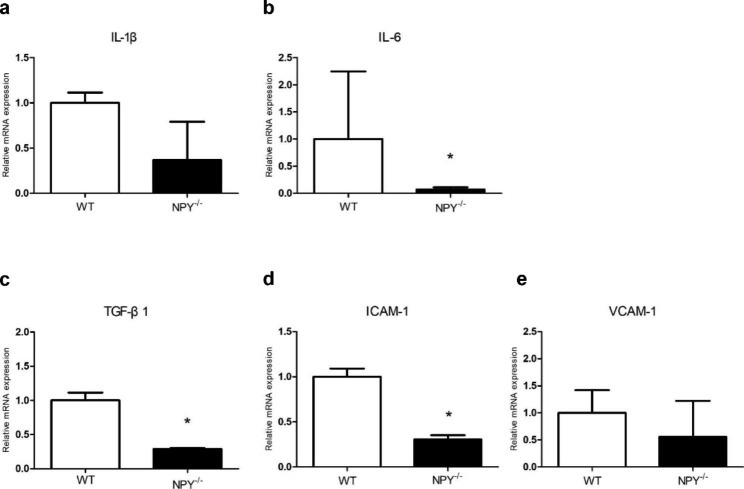
mRNA expression of several inflammatory markers and cell adhesion molecules in carotid arteries 21days after injury. The mRNA expression levels of IL-6 (b), TGF-β1 (c), and ICAM-1(d) were significantly decreased in NPY^−/−^ mice compared to those in WT mice. The expression of IL-1β and VCAM-1 mRNA in injured carotid artery of NPY^−/−^ mice tended to decrease (a, e). Values are mean ± SEM (n = 10), *p < 0.05

To further explore the molecular mechanisms by which NPY is regulated during neointimal formation, we assessed mRNA expression of several key inflammatory markers 21 days after carotid artery injury in WT mice. The mRNA expression of NPY^−/−^ mice was compared with that of WT mice 21 days after carotid artery injury. There was a tendency towards decreased mRNA expression of IL-1β in NPY^−/−^ mice left injured carotid artery (Fig. 3a). IL-6 and TGF-β1 mRNA expression in injured carotid artery of NPY^−/−^ mice was significantly lower than that of WT mice (0.069 ± 0.023, *p* < 0.05; 0.287 ± 0.008, *p* < 0.05, respectively) (Fig. 3b, c). Because cell adhesion molecules have been reported to be an important factor in restenosis, we also investigated ICAM-1 and VCAM-1 mRNAs in carotid arteries from these two mice at 21days after the injury. The mRNA expression of ICAM-1 in the injured carotid artery of NPY^−/−^ mice was significantly decreased (0.306 ± 0.031, *p* < 0.05) (Fig. 3d). Although the difference was not significant, there was a trend toward decreased expression of VCAM-1 mRNA in carotid artery lesions in NPY^−/−^ mice (Fig. 3e).

### Effects of NPY on inflammation mediator mRNA expression in cultured macrophages

**Fig. 4:**
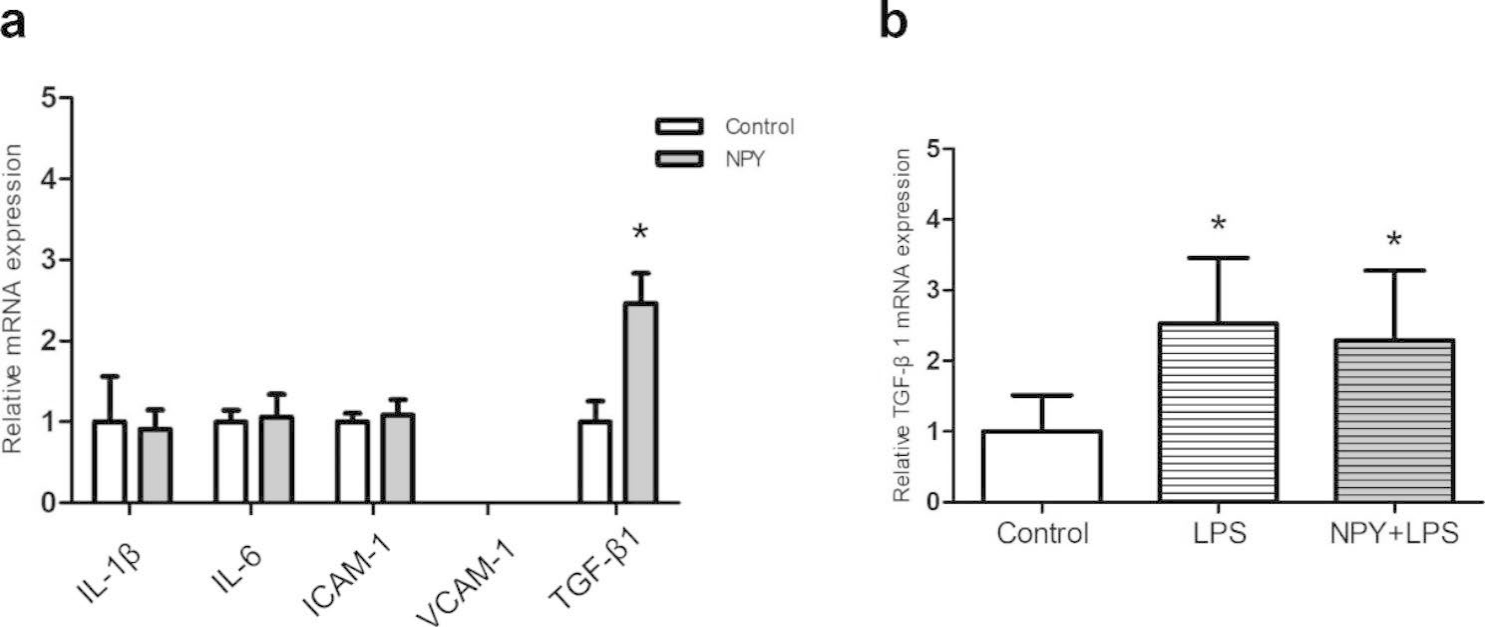
mRNA expression of several inflammation-related mediators in cultured macrophage cell line RAW264.7 cells. The mRNA expression level of TGF-β1 was significantly increased in 10^− 7^ M NPY treated RAW264.7 cells compared to that in control (a). The expression of TGF-β1 mRNA in LPS stimulated RAW264.7 cells was upregulated, with the presence of NPY it’s expression without further enhancement (b). Values are mean ± SEM (n = 4–5), *p < 0.05

After vascular injury, the monocyte/macrophages adhered and infiltrated into the subendothelium, thereby participating in local inflammation [26]. It has been reported that in artery neointima NPY and Y1 receptor were localized to macrophages, which indicated NPY may regulate the function of macrophages in artery disease [27]. Meanwhile, we found macrophages in neointima and mRNA expression of inflammatory mediators in injured artery were decreased in the absence of NPY, which also implied a regulatory role of NPY on macrophages. Hence, we utilized mouse macrophage cell line RAW264.7 cells to study the effects of NPY on mRNA expression of several inflammation-related mediators. Incubated RAW264.7 cells with 10^− 7^ M NPY for 12 h, RT-qPCR analysis showed that NPY could enhance the expression of TGF-β1 mRNA, but without influence on the mRNA expression of IL-1β, IL-6, ICAM-1 and VCAM-1(Fig. 4a). In the injured artery, with stimulation of local mechanisms, the infiltrated macrophages always become activated [28]. Therefore, we further studied the effects of NPY on TGF-β1 mRNA expression in activated macrophages. Incubated NPY pretreated RAW264.7 cells with 10ng/ml LPS for 12 h, we found that compared with control group, LPS stimulation could upregulate the expression of TGF-β1 mRNA in RAW264.7 cells, with the presence of NPY the expression of TGF-β1 mRNA in stimulated RAW264.7 cells without further enhancement (Fig. 4b). Meanwhile, we analyzed the effects of NPY on other inflammation mediators in stimulated RAW264.7 cells, and found the expression of IL-1β, IL-6, TNF-α, ICAM-1 and VCAM-1 were not influenced by 10^− 7^ M NPY incubation for 12 h (data not shown). Collectively, NPY could enhance TGF-β1 mRNA expression in unstimulated macrophages but not in LPS stimulated macrophages.

### Effect of NPY on macrophage proliferation

**Fig. 5:**
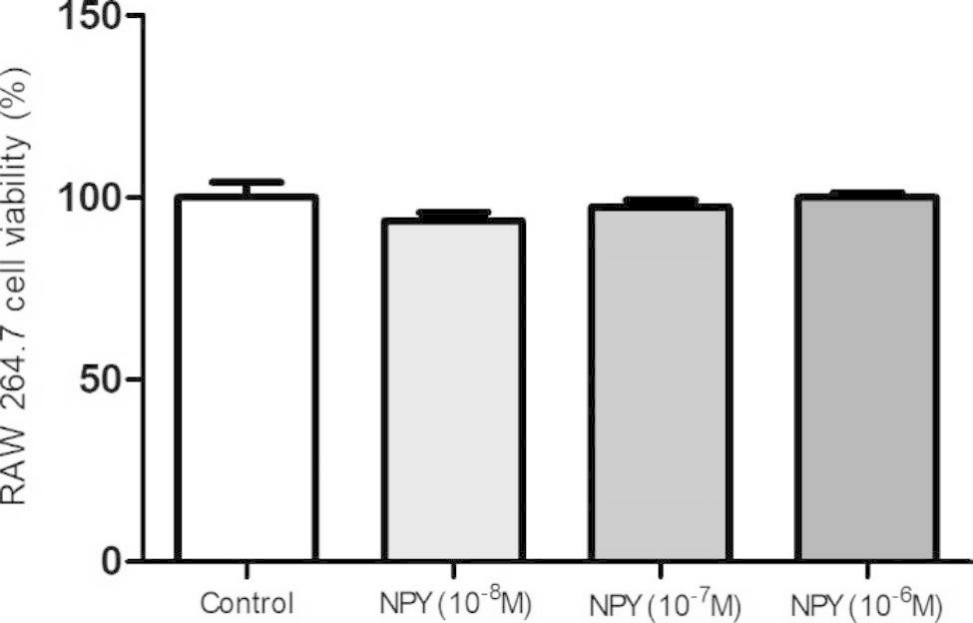
Effect of NPY on RAW264.7 cell proliferation. RAW264.7 cells were treated with graded doses of NPY (10^− 8^ M-10^− 6^ M) for 24 h and cell proliferation was determined using the CCK8 method, the number of cells was regarded as 100% in control group without sample treatment. Results are expressed as mean ± SEM of 3 sperate experiments run in quintuplicate

In hyperplastic intima of the artery, macrophages involved in multiple processes such as mediating inflammation, clearing damaged cells and local paracrine [29–31]. As shown in the above animal experiments, the carotid intima macrophage content was reduced in the absence of NPY, whether this decrease is related to the regulation of macrophage proliferation by NPY? To test this possibility, we performed a macrophage proliferation assay in vitro. RAW264.7 cells were treated with graded doses of NPY for 24 h and cell proliferation was determined by CCK8 method. Compared with control RAW264.7 cells, after 24 h of NPY incubation (10^− 8^ M, 10^− 7^ M and 10^− 6^ M), the cell viability did not change (Fig. 5), suggesting that these doses of NPY treated for 24 h didn’t affect macrophage proliferation. These results indicated that the decreased macrophages of carotid artery neointima in NPY^−/−^ mice may not result from NPY regulating macrophage proliferation.

## Discussion

In the present study, we demonstrated that NPY expression was upregulated in neointimal lesions, media and tissues around injured arteries in control mice, and vascular injury-induced neointima formation was significantly inhibited in the absence of NPY. This beneficial effect may be due to reduced macrophage content in neointimal lesions, as well as reduced mRNA expression of several critical inflammatory markers and cell adhesion molecules. Our findings suggest that the presence of NPY promotes neointima formation after arterial injury by regulating macrophage accumulation and local inflammatory responses.

Growing evidence indicates the importance of the autonomic nervous system in the pathogenesis of atherosclerotic disease [32, 33]. Although NPY is an important sympathetic neurotransmitter that has been implicated in restenosis and atherosclerosis [10, 21, 27, 34, 35], there are discrepancies in the literature and the underlying mechanisms are not fully understood. Inflammation plays an important role in the initiation and progression of restenosis [36, 37]. Studies have shown that NPY regulates the function of various inflammatory cells including monocytes, macrophages and lymphocytes in vitro [15, 38, 39].

Our finding that NPY deficiency inhibited neointima formation after arterial injury supported those earlier pharmacological observations in which NPY pellets placed next to injured arteries resulted in enhanced angioplasty induced neointima [12]. Meanwhile, along with the administration of NPY to damaged arteries using NPY^−/−^ mice could provide further information. Investigation of neointima formation while NPY deficiency or NPY up-regulated with pellets complement each other and could elucidate the role of NPY through counterregulatory mechanisms. Moreover, NPY deficiency could avoid the interference of diffusion properties and half-life of the recombinant protein, and better simulated the physiological process. At the same time, we further demonstrated that NPY promoted neointima formation by regulating the inflammatory response of injured arteries.

We also found that compared with WT mice, NPY^−/−^ mice had lower macrophages content in neointimal lesions. Furthermore, when NPY was deficient, several critical inflammation-related mediators (IL-6, TGF-β1, ICAM-1) in the injured artery were inhibited, indicating that NPY controlled this process through affecting the expression of these markers. Previous studies have shown that IL-6 acted on smooth muscle cells and promoted neointimal hyperplasia by inducing a synthetic phenotype of VSMCS through JAK/STAT signaling [40]. TGF-β signaling could promote neointima formation by regulating key aspects: inflammation, chemotaxis, fibrosis, proliferation, and extracellular matrix production [41]. ICAM-1 was fundamental in inflammatory processes after vascular intervention. Vascular injury led to an enhanced expression of ICAM-1 on the luminal surface, which promoted the adhesion and migration of neutrophils, lymphocytes and monocytes, further causing acute and chronic inflammatory responses [42].

Our in vitro mechanistic data supported this concept since we found 10^− 7^ M NPY could promote TGF-β1 mRNA expression in macrophage cell line RAW264.7 cells. Meanwhile, NPY had no effect on macrophage proliferation in vitro, indicating the decreased macrophages of carotid artery neointima in NPY^−/−^ mice was not result from NPY regulating macrophage proliferation, may derived the decreased adherence and infiltration of macrophages at injured artery. It should be pointed out that NPY of the injured artery may not only derived from sympathetic nerve endings. Circulating platelets also contained abundant NPY, this peptide was stored in platelet granules and released during platelet activation and aggregation [43]. During artery intervention procedures, endothelial denudation and subsequent exposure of collagen result in the activation and adherence of platelets, thereby inducing the release of NPY to injured position. Based on our findings, we hypothesized that the mechanism of NPY-regulated restenosis was as follows: In the presence of vascular injury (i.e., endothelial injury, angioplasty or stent implantation), NPY was released from sympathetic nerve endings and locally adhered platelets and accumulated in the injured artery. As a result, increased NPY promotes the expression of ICAM-1 and VCAM-1 in damaged arteries, which in turn led to increased adhesion and migration of inflammatory cells, thereby promoting neointima formation. Thus, blocking the action of NPY may have significant potential to attenuate restenosis. Although we have shown that NPY affects neointima formation through an inflammatory pathway, but it is possible that other pathways and cascades also contribute to this phenotype. Matrix metalloproteinases (MMPs) were a kind of important mediators of neointima formation follow vascular injury. Xiao and colleagues found compared with ApoE^−/−^ mice, MMP8^−/−^ApoE^−/−^ mice had decreased neointima formation and fewer proliferating cells in neointima after carotid artery injury [44]. MMP8, also known as neutrophil collagenase, was once thought to be expressed exclusively in neutrophil cells, but more recent researches have shown endothelial cells, VSMCs and macrophages in atherosclerosis express MMP8 [45]. At present the effect of NPY on MMP8 expression was poorly understood, only the promotion of NPY on macrophages MMP8 expression was discovered. However, the regulatory effect of NPY on MMP8 expression in neutrophil cells, endothelial cells and VSMCs (the major source of MMP8 in injured artery) has not been investigated. It is noteworthy that NPY stimulates food intake and inhibits energy expenditure, leading to obesity [46]. Therefore, it plays a critical role in the regulation of energy homeostasis [47]. Recently, strategies targeting the NPY signaling pathway have been explored for obesity and diabetes interventions in animal studies, and some exciting metabolic benefits have been reported [48–52]. Since obesity and diabetes are key risk factors for cardiovascular disease, blocking the effects of NPY would not only have beneficial effects on local restenosis after arterial injury, but would also improve energy metabolism throughout the body, this reduced fat, a major potential cause of arteriosclerosis development and subsequent neointimal formation.

Although our study revealed a critical role for NPY in neointimal development following vascular injury, certain limitations should be noted. First, the FeCl_3_ carotid arterial injury mouse model cannot entirely resemble the pathophysiology of human post-PCI restenosis. Other pre-clinical large animal models (e.g. porcine PTCA model, porcine coronary artery stenting model) might be needed for exploring the precise role of NPY in restenosis. Second, only male mice were used in this research. Although most of the basic researches for neointima formation apply male animals, it has been reported in clinical research that women presented a 23% reduction of the risk of restenosis, which means gender may play a role in restenosis. Whether the regulation of NPY on neointima formation which was discovered in the present study feasible in female was uncertain. Moreover, the lipid profile of mice was different from humans’, the atherogenic diet feeding may not exactly mimic human hypercholesterolemia. Thus, further studies are needed to validate the role of NPY in neointima formation in clinical settings. Third, we observed that NPY deficiency downregulated the expression of several key inflammatory factors and adhesion molecules in injured carotid arteries; but the exact mechanism of the intracellular regulation and interaction among these differentially expressed factors remains to be further investigated.

## Conclusions

In conclusion, we have showed that NPY plays a critical role in the development of neointima formation following vascular injury, and that lack of NPY attenuates scar tissue formation following vascular injury. This study provides a better understanding of neointima formation and highlights the potential that blocking NPY signaling may have beneficial effects in atherosclerosis and restenosis.

## Data Availability

The data used to support the findings of this study are available from the corresponding author upon request.

## Abbreviations

FeCl_3_: Ferric chloride
ICAM-1: Intercellular adhesion molecule-1
IL-6: Interleukin-6
LPS: Lipopolysaccharide
NPY: Neuropeptide Y
OCT: Optimal cutting temperature compound
PCA: Percutaneous coronary angioplasty
PCI: Percutaneous coronary intervention
RT-qPCR: Real-time quantitative PCR
TGF-β1: Transforming growth factor-β1
VSMCs: Vascular smooth muscle cells
WT: Wild-type
